# Evaluation of a group-based sensorimotor intervention programme to improve Chinese handwriting of primary school students

**DOI:** 10.1016/j.heliyon.2022.e12554

**Published:** 2022-12-23

**Authors:** Cecilia W.P. Li-Tsang, Tim M.H. Li, C.N. Yang, Phoebe P.P. Cheung, K.Y. Au, Y.P. Chan, K.Y. Cheung, K.H. Ho, K.W. Kwok, Howard W.H. Leung

**Affiliations:** a Faculty of Health and Social Sciences, The Hong Kong Polytechnic University, Hong Kong; b Department of Psychiatry, Faculty of Medicine, The Chinese University of Hong Kong, Hong Kong; c Department of Computer Science, City University of Hong Kong, Hong Kong; d Yew Chung College of Early Childhood Education, Hong Kong; e Department of Rehabilitation Sciences, The Hong Kong Polytechnic University, Hong Kong

**Keywords:** Handwriting, Sensorimotor, Chinese, Intervention, Primary school students

## Abstract

**Objective:**

Sensorimotor performance is influential in Chinese handwriting, but few studies have examined the efficacy of sensorimotor-based interventions on Chinese handwriting among primary school students with poor handwriting performance. The study aims to evaluate a sensorimotor-based intervention to improve handwriting in the mainstream primary schools.

**Methods:**

This study adopted a two-group pretest-posttest design. An 8-session group-based sensorimotor intervention was delivered to school-aged children (mean age = 8.1, 68% male). Group A had 2 sessions every week, while Group B had 4 sessions every week. Analysis of variance with repeated measures was used to test the effects.

**Results:**

The intervention had a significant time effect (*p* < .05) in terms of improving handwriting process (*d* = 0.33–1.10), manual dexterity (*d* = 0.57), visual memory (*d* = 0.70), visual-spatial perception (*d* = 0.37), and motor and postural skills (*d* = 0.73). The effect sizes ranged from medium to large. For the handwriting process, time per character had a significant group × time interaction, with post hoc analysis showing that Group A had a significantly large effect (*d* = 1.89, *p* < .001) while Group B did not.

**Conclusions:**

The group-based sensorimotor intervention programme appeared to show improvements in students with fair skills in writing Chinese characters. It appears that the effect is better if the training sessions are spaced out in one month rather than intensively conducted within two weeks. It might be related to more involvement from parents, and students need more time for practice after the training sessions.

## 1 Introduction

Children acquire handwriting skills at the age of 5–6 for expressing, communicating, and recording ideas, and over 50% of their school time is spent on handwriting tasks [1]. It is estimated that around 10–30% of school-aged children have handwriting difficulties [2], which is one of the most common reasons for referral for occupational therapy intervention [3]. Handwriting problem is detrimental to students' academic performance, particularly when they are asked to sit in tests and examinations which require fast handwriting speed [4]. Poor academic performance can lead to a long-term negative impact on students' self-esteem, academic achievement, behavior, and attitude [5]. Many teachers and parents adopt a drill-and-practice method to improve children's handwriting performance, which is suggested to be effective, but it might be boring to children, resulting in a lack of motivation to write [6,7]. On the other hand, occupational therapists have attempted to provide interventions targeting on children's sensorimotor performance, given the strong associations of motor control and visual perceptual skills with handwriting. With the design of the training using play and games, therapists aim to enhance children's sensorimotor development and hence improve their handwriting performance. Few studies, however, have evaluated the efficacy of these interventions on Chinese handwriting in children [8].

Handwriting performance is often evaluated based on handwriting speed and accuracy; children's abilities to write are based on various sensorimotor skills, namely, fine motor control, visual perception, oculomotor control, and visual-motor integration [1,9,10]. Motor skills for controlling the coordinated arm, hand, and finger movements are found to correlate with handwriting speed [11]. It is also reported that better fine motor control could result in more consistent pen pressure and hence better handwriting stability. Visual perceptual skills such as space and size discrimination, visual orientation, and distinguishing graphic forms could facilitate character recognition [5]. Oculomotor skills, including saccades and pursuits are vital for visual tracking to capture useful information, thus reducing the time for copying [10]. The ability to integrate visual perceptual information and motor movement is related to handwriting legibility performance [12]. Systematic reviews on sensorimotor-based handwriting interventions reported small to medium effect sizes in improving handwriting legibility [6,8]. The effect sizes for handwriting speed highly varied across studies.

The basic writing unit in Chinese is a character, which is morpho-syllabic, that usually consists of one syllable and one morpheme [13,14]. Unlike the alphabetic writing system of English, logographic Chinese characters have inconsistent phonology and orthography which demands more memorization during the learning and acquisition of Chinese. English requires writers to produce written words with a fixed set of 26 letters on horizontal grid lines [12], while Chinese characters are organized in a square construction with a compilation of strokes and radicals [14]. More than 600 orthographic components (radicals and strokes) are combined to form thousands of Chinese characters [15]. If English words are written with wrong spacing or wrong alphabetic order, teachers may still be able to guess the correct words. However, with wrong strokes or radical positions and spacing, Chinese characters can have different meanings. The complex geometric figuration and arrangement of strokes in a squared area make Chinese more demanding than English in handwriting proficiency and requires more visual memory, visual perceptual and visual motor skills [1].

This study evaluated a group-based sensorimotor intervention programme for improving Chinese handwriting in primary school students, while most previous studies evaluated sensorimotor interventions for enhancing English handwriting. Based on the motor learning theory, the experience of a set of associated practices can lead to relatively permanent changes in the capacity for movement. To make a permanent change in handwriting, the key principle of motor learning is practicing a task alternating with periods of rest and feedback [16]. Furthermore, training in small groups, similar to school and classroom environments, provides students an opportunity to request or give mediation to their peers according to sociocultural theory [17]. In addition, previous studies on a group of four students of six training sessions and a preliminary trial on a group of six students with eight sessions appeared to have promising results from the test results [18,19]. Thus, the selection of the eight sessions of training was adopted with two or four sessions of training per week after compromising the feasibility and availability of the students, parents, and school arrangements during the COVID-19 pandemic. More scientific evidence is needed to explore whether these sensorimotor-based interventions could improve Chinese handwriting, given that sensorimotor performance can be even more influential to Chinese handwriting. For example, visual-spatial perception is essential for the correct spatial arrangement of Chinese characters due to their logographic nature.

The effect sizes for handwriting speed varied across previous studies, which might be due to lack of objective, quantitative evaluation methods for assessing handwriting abilities. Handwriting performance is difficult to measure objectively, and a lot of handwriting assessments are developed in the forms of questionnaires. Some use copying speed as the criteria for assessment, and they use English writing as a template for copying. For Chinese handwriting assessment, Tseng's handwriting test uses a checklist and a copying test to determine the speed [20]. However, it remains difficult to assess the legibility and accuracy of the writing. Thus, in this study, we would like to assess both the handwriting process and product (speed and accuracy) more quantitatively and objectively. As our targeted students are from Hong Kong, we have to find a handwriting assessment tool to screen out students with handwriting difficulties. A norm reference has been obtained by using the computerized handwriting assessment tool, the Smart Handwriting Analysis and Recognition Platform (SHARP) [21]. Hence, this study also aims to evaluate if this SHARP system can discriminate students with handwriting problems in mainstream primary schools. This remains important as the SHARP system can help to identify the group of students we intend to support. The SHARP system could accurately measure the speed and total time and further discriminate the in-air time and ground time to see if the child has problems tracking or writing the words. SHARP could also find out the writing errors, namely missing and additional strokes, wrong stroke sequences, out of grids and so forth. Therefore, the handwriting performance of students joining the training programme could be measured both qualitatively and quantitatively. In the study, an 8-session of sensorimotor intervention was organized with one group (Group A) having 2 sessions per week for 4 weeks, while the second group (Group B) had training 4 sessions per week in 2 weeks. Their handwriting performance was measured and compared before and after the intervention to find out if the total length of intervention would affect the outcomes.

## 2 Material and methods

### 2.1 Study design and participants

**Fig. 1:**
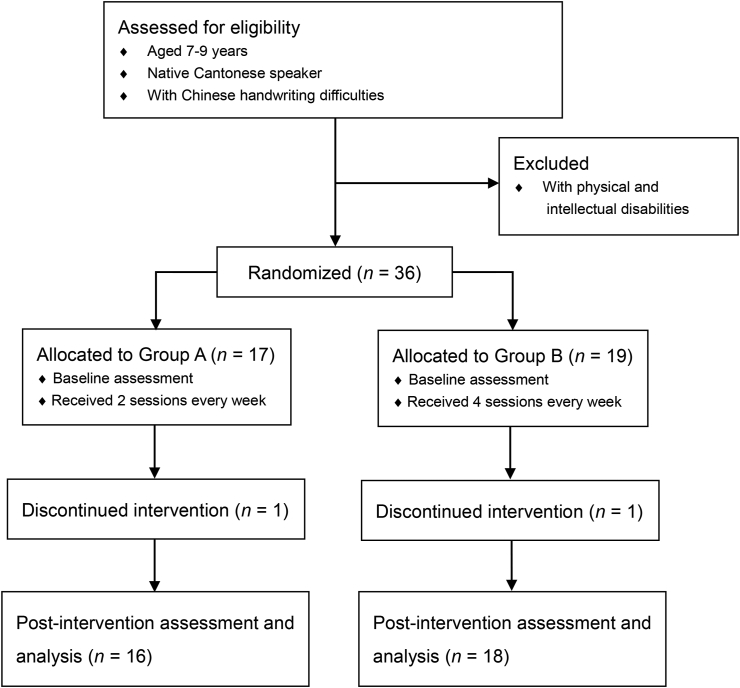
Study flow diagram.

This study adopted a two-group pretest-posttest design. An 8-session group-based sensorimotor intervention was delivered to school-aged children from March to August 2021. The rationale for choosing an 8 sessions of group intervention was based on previous studies on training motor skills for children with developmental coordination disorders (DCD) [19]. Group training appears to be more cost-effective, and it also allows peer group learning and support. The group training sessions were conducted at the school after class, thus students joining the programme were regarded as joining an activity programme rather than having individual students being taken out from the classroom for training. The arrangements for teachers, parents, students, and therapists would be more convenient and efficient. The first group of students would have 2 sessions every week (Group A), while Group B had 4 sessions every week for two weeks. Participants were randomly assigned to the groups. Pre- and post-assessments were administered to evaluate participants’ handwriting and sensorimotor performance. All participants were assessed within two weeks before the first session and after the last session. The study flow is illustrated in Fig. 1.

Grade 2–3 students from local mainstream schools were invited to participate in the study using convenience sampling. The inclusion criteria included (1) aged 7–9 years, (2) native Cantonese speaker, (3) with Chinese handwriting difficulties reported by teachers and parents, and (4) results from SHARP evaluation showing one standard deviation (*SD*) below average in any of the parameters of the handwriting process (including ground time, air time, speed and *SD* of writing time per character) and handwriting product (including out of grid, missing stroke, concatenated stroke, wrong stroke, reversed stroke, and recognized character) [21], and (5) assessed by occupational therapists to have sensorimotor difficulties. Occupational therapists assessed the potential participants to evaluate their handwriting and sensorimotor performance. Students with diagnosis of specific learning difficulties (SpLD) or other diagnosis of developmental delay were excluded from the study.

The study was approved by the Departmental Research Committee of the Department of Rehabilitation Sciences at the Hong Kong Polytechnic University (Reference number: HSEARS20210209003). Written consent was obtained from all participants' parents prior to the intervention, and information sheets were given regarding the intervention details.

### 2.2 The sensorimotor intervention programme

The sensorimotor intervention programme is consisted of 8 group-based sessions with each session lasting for 80–90 min. Before the training programme, a briefing session was arranged to inform parents of the contents of the training. The programme was composed of four themes of training, namely, training of fine motor skills, visual perceptual skills, visual motor integrative skills, and ocular motor skills, with two sessions for each theme. In each session, there would be a warm-up activity on general body movements followed by three to four game based activities on the selected theme. These activities were specially designed by the occupational therapists to enhance the fine motor, visual motor integration, visual perception or ocular motor control. within group. These game based activities were conducted in group format with competitive nature to arouse participants’ engagement in the activities. There were also illustrations of the activities selected for each theme of training as shown in Table 1. After each training session, parents were informed of the theme of the training, and they were given a worksheet to encourage their children to engage in particular training activities at home and were reminded by communication software. For example, the theme was on fine motor skills training, then parents were asked to engage their children in some games which involved the use of fine motor skills.

**Table 1:** Outline of the themes and activities in the 8-session intervention.

Session	Warm up activity (15–20 min)	Theme	Theme-based activities (50–60 min)
1	Vestibular, proprioceptive, tactile stimulation and finger activities.	Visual-motor integration	Tracing lines, origami
2			String figure, card pyramid
3		Oculomotor control	Bean bag games, oculomotor worksheets (look & find)
4			Bean bag games, oculomotor worksheets
5		Fine motor control	Cutting and pasting, paper basketball game
6			Paper-tear painting, finger maze
7		Visual perception	Number link puzzles, long picture puzzles
8			Mirror drawing, connect the dots game

### 2.3 Measures

The Smart Handwriting Analysis and Recognition Platform (SHARP) was used to assess the Chinese handwriting performance of primary school students [21]. Participants were required to copy 90 Chinese characters on a digital handwriting tablet. For the handwriting process, ground time (on-paper time), air time (on-air time), air/ground time ratio, time per character, writing speed (character per minute), *SD* of the writing time per character, pen-tip pressure, and *SD* of pressure were recorded. The time was measured in seconds. The parameters of the handwriting product are measured by character size (mm^2^) and the percentage of characters written out of grid, recognized, and with the wrong stroke (WS), additional stroke (AS), missing stroke (MS), concatenated stroke (CS), reverse stroke (RS), and wrong stroke sequence (WSS).

The Handwriting Assessment Checklist (HAC) has 10 questions to assess three domains of handwriting, including the writing process, writing product, physical and emotional well-being. The checklist was rated by participants' parents in the study. The total score of the HAC ranged from 0 to 40. A higher score represents a decrease in handwriting proficiency. The checklist has acceptable internal consistency with the Cronbach's alpha of 0.80 and adequate test-retest reliability (r = 0.84) [1].

The Bruininks-Oseretsky Test of Motor Proficiency – 2nd Edition (BOT) was used to measure motor skills [22]. BOT is a standardized, norm-referenced assessment for children and adolescents aged 4 to 21 in four fine motor areas including Fine Motor Precision (FMP), Fine Motor Integration (FMI), Manual Dexterity (MD), and Upper Limb Coordination (ULC) were selected for fine motor function testing. A higher score indicates a greater function in motor control. The subtests of BOT-2 have good inter-rater reliabilities (>0.90) [23].

Test for Visual Perceptual Skills – 4th Edition (TVPS) was used for visual perceptual skills testing [24]. TVPS-4 is a standardized, norm-referenced assessment for motor-free visual perceptual skills for children and young adolescents aged 5 to 21. There are seven subtests, including Visual Discrimination (DIS), Visual Memory (MEM), Visual-Spatial Relationships (SPA), Form Constancy (CON), Visual Sequential Memory (SEQ), Visual Figure-Ground (FGR), and Visual Closure (CLO). Participants were instructed to view a picture with multiple choices and select the correct answer. A higher score indicates a greater function in visual perception. TVPS-4 demonstrated good reliability. Its internal-consistency reliability is acceptable with the average Cronbach's alpha value for overall TVPS-4 subtests as 0.94. It has adequate test-retest reliability with an average corrected correlation score for overall subtests of 0.97 [24].

The Developmental Eye Movement Test – 2nd Edition (DEM) was used to evaluate oculomotor control [25]. The DEM is a standardized, norm-referenced assessment for oculomotor functions related to reading for individuals aged 6 to 13. It consists of a vertical subtest (DEM-V) and a horizontal subtest (DEM-H). Participants were instructed to read the digital number displayed in a vertical array in DEM-V. Participants were asked to read the digital number displayed in a horizontal array with uneven distribution without the assistance of a finger in DEM-H. The ratio (DEM-R) was calculated. A higher DEM-R and a higher DEM-H score indicate more oculomotor dysfunction. DEM-2 demonstrates good reliability due to the high test-retest reliability of vertical time and adjusted horizontal time and moderate to high for ratios and errors [26].

The Clinical Observation of Motor and Postural Skills – 2nd Edition (COMPS) is an individually administered standardized test to screen for developmental motor coordination problems for individuals aged 5 to 15 [27]. COMPS is made up of six items including slow movements, rapid forearm rotation, finger-nose touching, prone extension posture, asymmetrical tonic neck reflex, and supine flexion posture. A weighted total score is calculated. A higher score indicates better motor and postural performance. The test demonstrates high test-retest reliability (*r* = .92) and excellent inter-rater reliability with a correlation coefficient of 0.87 [27].

A self-constructed questionnaire was used to collect parent feedback after the training sessions. There are 7 items in the questionnaire, mainly on their feedback on the handwriting assessment (SHARP) and intervention programme using a 5-point response format.

### 2.4 Statistical analyses

Means and *SD* for the continuous variables were presented, while numbers and percentages for the categorical variables were shown. Demographic differences between Group A and Group B were analyzed using the Chi-square test and *t*-test. Baseline differences between the groups were investigated using the *t*-test. Analysis of covariance (ANCOVA) with repeated measures was used to test the group (between-subject factor), time (within-subject factor), and group × time interaction effects of the outcomes (including handwriting process and product, BOT, TVPS, DEM, COMPS, and HAC) between Group A and Group B. The analyses were adjusted for gender. For a significant interaction, post hoc comparisons between the pretest and posttest in the groups were conducted using a *t*-test with the Bonferroni correction. A Cohen's d effect size for each significant outcome was calculated, where *d* = 0.2, 0.5, and 0.8 correspond to small, medium, and large effect sizes. The associations between the change scores of sensorimotor skills (independent variables) and children's handwriting outcomes (dependent variables) after the interventions were examined using simple linear regression. The analyses were also adjusted for gender. The coefficient and 95% confidence interval of the significant associations were presented. All statistical analyses were performed using statistical software R for Windows (R version 4.1.0). A p-value <.05 was considered statistically significant.

## 3 Results

### 3.1 Participants

36 Grade 2–3 students from mainstream schools participated in the intervention. With one participant dropping out from each group, there were 16 and 18 participants who completed the intervention programme in Group A and B respectively. All participants were aged 7–9 years (mean age = 8.19, *SD* = 0.61). More males (*n* = 23, 68%) had participated in the intervention than females (*n* = 11, 32%). Thus, the ANOVA analyses were adjusted for gender. Only one participant in Group B was left-handed. All other participants were right-handed. Table 2 shows that there were no statistically significant demographic differences between Group A and Group B. Table 3 reveals the handwriting and sensorimotor performance of participants. The participants had a longer air time than the ground time during handwriting. Among six handwriting errors, WS and AS were the most common errors among the participants.

**Table 2:** The demographic differences between Group A and Group B.

	Group A (*n* = 16)	Group B (*n* = 18)	*P*
	Mean (*SD*)/*n* (%)		
Age	8.17 (0.60)	8.46 (0.61)	0.22
Gender			0.81
Male	10 (63)	13 (72)	
Female	6 (37)	5 (28)	
Handedness			>.99
Right-handed	16 (100)	17 (94)	
Left-handed	0 (0)	1 (6)

**Table 3:** The outcomes of Group A and Group B in pretest and posttest.

	Overall (*n* = 34)		Group A (*n* = 16)		Group B (*n* = 18)		Baseline difference
Outcomes	Pretest	Posttest	Pretest	Posttest	Pretest	Posttest	
	Mean (*SD*)/*n* (%)						*p*
Handwriting process							
Air time (s)	560.83 (195.80)	460.71 (154.54)	565.33 (178.01)	455.17 (124.71)	555.77 (219.95)	466.94 (186.65)	0.89
Ground time (s)	340.86 (99.42)	308.76 (94.19)	328.93 (106.66)	310.38 (104.76)	354.28 (92.14)	306.94 (84.11)	0.46
Total time (s)	901.69 (256.40)	769.55 (199.69)	894.26 (246.96)	765.54 (189.87)	910.05 (274.52)	774.06 (216.39)	0.86
Air/ground time ratio	1.72 (0.58)	1.58 (0.58)	1.81 (0.58)	1.58 (0.53)	1.60 (0.58)	1.59 (0.65)	0.31
Time per character (s)	10.05 (2.87)	6.98 (2.70)	9.98 (2.77)	8.58 (2.15)	10.14 (3.06)	5.19 (2.07)	0.88
SD of time per character	5.48 (1.78)	4.80 (1.45)	5.61 (1.50)	4.92 (1.44)	5.34 (2.10)	4.67 (1.50)	0.67
Writing speed	6.56 (2.25)	7.52 (2.29)	6.49 (1.85)	7.43 (1.87)	6.64 (2.70)	7.62 (2.74)	0.85
Average pressure	1594.73 (204.81)	1624.18 (172.70)	1586.28 (219.59)	1586.75 (165.42)	1604.23 (193.53)	1666.29 (176.14)	0.80
SD of pressure	489.43 (58.32)	478.47 (52.95)	474.07 (53.74)	489.27 (50.88)	506.71 (60.06)	466.32 (54.19)	0.11
Handwriting product							
Character size (mm^2^)	113.25 (25.70)	128.58 (31.75)	113.87 (29.84)	136.45 (38.00)	112.55 (21.04)	119.74 (20.57)	0.88
Out of grid (%)	29.58 (21.33)	40.23 (19.20)	31.36 (23.40)	42.16 (18.89)	27.57 (19.28)	38.06 (19.91)	0.61
Recognized character (%)	93.09 (7.88)	92.50 (7.31)	93.61 (8.54)	91.11 (7.19)	92.50 (7.30)	94.06 (7.35)	0.69
Wrong stroke (%)	33.12 (16.45)	39.93 (19.56)	35.07 (17.73)	42.30 (21.57)	30.93 (15.14)	37.26 (17.32)	0.47
Additional stroke (%)	33.42 (14.71)	34.18 (16.18)	32.09 (13.90)	35.48 (15.08)	34.93 (15.88)	32.71 (17.71)	0.59
Missing stroke (%)	18.29 (13.67)	16.45 (10.20)	25.26 (12.77)	17.74 (10.37)	10.44 (10.10)	14.99 (10.13)	<.001∗∗∗
Concatenated stroke (%)	30.20 (13.12)	32.92 (17.29)	31.49 (10.73)	32.05 (16.31)	28.75 (15.62)	33.90 (18.82)	0.56
Reversed stroke (%)	6.07 (8.96)	5.22 (6.20)	6.83 (10.85)	5.22 (5.62)	5.21 (6.45)	5.22 (6.98)	0.60
Wrong stroke sequence (%)	25.49 (12.68)	23.75 (11.14)	23.28 (11.90)	24.03 (12.75)	27.97 (13.44)	23.42 (9.41)	0.29
HAC	23.41 (9.21)	25.90 (9.14)	19.22 (8.15)	23.00 (7.69)	28.79 (7.76)	28.62 (9.77)	0.002∗∗
Sensorimotor skills							
BOT							
FMP	13.47 (3.93)	13.85 (4.02)	11.94 (3.93)	12.83 (3.28)	15.19 (3.25)	15.00 (4.56)	0.01∗
FMI	16.15 (4.21)	16.97 (5.45)	14.22 (3.75)	14.83 (4.19)	18.31 (3.68)	19.38 (5.81)	0.003∗∗
MD	14.68 (3.92)	16.68 (3.03)	15.67 (4.07)	17.17 (3.57)	13.56 (3.54)	16.12 (2.28)	0.12
ULC	10.32 (4.20)	9.91 (3.92)	9.61 (4.34)	10.28 (4.87)	11.12 (4.03)	9.50 (2.56)	0.30
TVPS							
CLO	9.85 (3.08)	10.59 (2.60)	10.17 (3.02)	10.33 (2.97)	9.50 (3.20)	10.88 (2.16)	0.54
MEM	10.15 (2.32)	11.79 (2.35)	9.94 (2.92)	11.94 (2.48)	10.38 (1.46)	11.62 (2.25)	0.59
SPA	11.79 (2.51)	12.71 (2.42)	12.06 (2.69)	13.06 (2.34)	11.50 (2.34)	12.31 (2.52)	0.52
CON	10.38 (3.21)	11.18 (3.33)	10.06 (3.83)	11.22 (3.47)	10.75 (2.41)	11.12 (3.26)	0.53
SEQ	11.65 (2.33)	11.79 (3.18)	11.67 (2.38)	11.78 (2.37)	11.62 (2.36)	11.81 (3.99)	0.96
FGR	10.27 (2.48)	10.27 (3.14)	10.11 (2.76)	11.11 (2.68)	10.44 (2.19)	9.31 (3.42)	0.70
DEM							
DEM-V score	50.68 (11.35)	51.21 (11.60)	51.72 (10.82)	54.61 (11.92)	49.50 (12.16)	47.39 (10.28)	0.58
DEM-H score	66.61 (16.45)	62.50 (17.43)	66.58 (14.34)	63.39 (16.64)	66.64 (19.03)	61.49 (18.78)	0.99
DEM-R	1.33 (0.26)	1.22 (0.20)	1.30 (0.24)	1.16 (0.15)	1.35 (0.29)	1.29 (0.22)	0.58
COMPS	1.56 (1.31)	2.37 (0.86)	1.76 (1.12)	2.31 (0.93)	1.33 (1.50)	2.44 (0.81)	0.36

Adjusted for gender; ∗<0.05, ∗∗<0.01, ∗∗∗<0.001.

### 3.2 Effect of the sensorimotor intervention programme

**Fig. 2:**
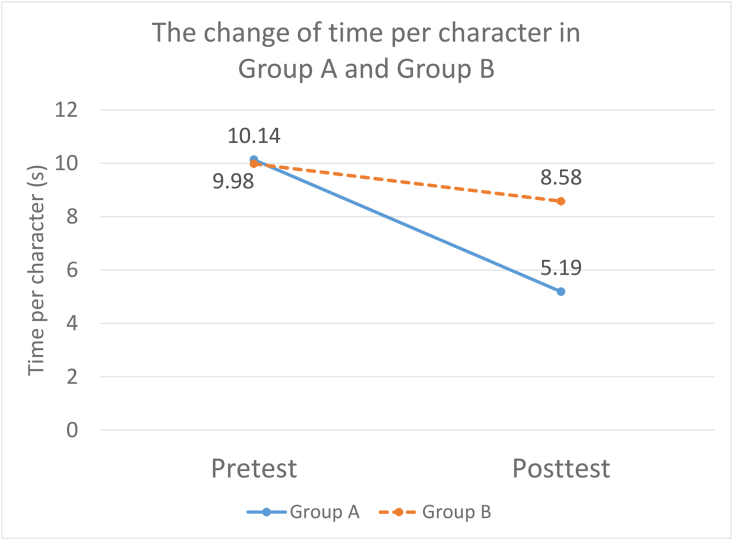
The change of time per character in Group A and Group B.

There were baseline differences between the two groups in terms of MS, HAC, FMP, and FMI as shown in Table 3. Group A had a poorer performance in MS, FMP, and FMI than Group B. Group B had a higher HAC than Group A reflecting. Table 4 illustrates the effect of the intervention. The intervention (including Group A and Group B) had a significant time effect (*p* < .05) on the outcomes including air time (*d* = 0.57), ground time (*d* = 0.33), total time (*d* = 0.58), time per character (*d* = 1.10), speed (*d* = 0.42), size (*d* = 0.53), MD (*d* = 0.57), MEM (*d* = 0.70), SPA (*d* = 0.37), and COMPS (*d* = 0.73). The effect sizes ranged from medium to large. Among the outcomes, participants showed the largest improvement in time per character. Nevertheless, time per character had a significant group × time interaction, *F* (1, 31) = 22.88, *p* < .001, with post hoc analysis showing that Group A had a significant large effect (*d* = 1.89, *p* < .001) while Group B did not. Fig. 2 illustrates the significant interaction effect by comparing time per character of Group A and Group B between pretest and posttest. More out of grid (*d* = 0.53) was observed in participants after the intervention. There was a marginally significant effect on DEM-R (*p* = .06).

**Table 4:** The group, time, and interaction effects of the interventions.

Outcomes	Group effect		Time effect		Interaction effect		Post hoc analysis	
							Group A (Posttest-Pretest)	Group B (Posttest-Pretest)
	*p*		*p*		*p*		*p*	*p*
Handwriting process								
Air time	0.99		<.001	∗∗∗	0.70			
Ground time	0.84		0.04	∗	0.29			
Total time	0.94		0.001	∗∗	0.88			
Air/ground time ratio	0.68		0.09		0.10			
Time per character	0.05	∗	<.001	∗∗∗	<.001	∗∗∗	<.001∗∗∗	0.10
SD of time per character	0.57		0.13		0.91			
Writing speed	0.74		0.005	∗∗	0.94			
Average pressure	0.53		0.36		0.18			
SD of pressure	0.69		0.28		0.02	∗	0.11	0.39
Handwriting product								
Character size	0.25		0.04	∗	0.22			
Out of grid	0.61		0.03	∗	0.94			
Recognized character	0.63		0.92		0.19			
Wrong stroke	0.43		0.05		0.85			
Additional stroke	0.97		0.86		0.38			
Missing stroke	0.01	∗	0.99		0.01	∗	0.21	0.12
Concatenated stroke	0.98		0.27		0.38			
Reversed stroke	0.75		0.37		0.44			
Wrong stroke sequence	0.48		0.65		0.17			
HAC	0.04	∗	0.25		0.09			
Sensorimotor skills								
BOT								
FMP	0.03	∗	0.41		0.35			
FMI	0.004	∗∗	0.51		0.65			
MD	0.11		0.002	∗∗	0.32			
ULC	0.72		0.19		0.04	∗	0.37	0.67
TVPS								
CLO	0.95		0.16		0.37			
MEM	0.88		<.001	∗∗∗	0.35			
SPA	0.43		0.02	∗	0.73			
CON	0.74		0.09		0.38			
SEQ	0.91		0.59		0.99			
FGR	0.36		0.62		0.04	∗	0.28	0.28
DEM								
DEM-V score	0.12		0.54		0.05			
DEM-H score	0.67		0.18		0.60			
DEM-R	0.18		0.06		0.42			
COMPS	0.68		<.001	∗∗∗	0.14			

Adjusted for gender; ∗<0.05, ∗∗<0.01, ∗∗∗<0.001.

### 3.3 Associations between sensorimotor skills and handwriting performance

Table 5 presents the significant associations between the change scores of sensorimotor skills and children's handwriting outcomes after the interventions. The improvement of COMPS was associated with multiple handwriting outcomes including reduced writing time, lower variation in writing speed, increased writing speed, and fewer stroke errors (i.e., reversed stroke and wrong stroke sequence). The improvement of SEQ was associated with reduced *SD* of time per character. The improvement of DEM-V was associated with reduced time per character, fewer missing strokes, and a higher HAC score. The improvement of DEM-R was associated with higher pen pressure.

**Table 5:** Significant associations between the change scores of sensorimotor skills and children's handwriting outcomes after the interventions.

Sensorimotor skills		Handwriting performance		*β* (95% *CI*)	*p*
	Post – pre		Post – pre		
	Mean (*SD*)		Mean (*SD*)		
TVPS MEM	1.65 (2.52)	Concatenated stroke	2.72 (14.40)	−2.19 (−4.14, −0.23) ∗	.03
TVPS SEQ	0.15 (3.14)	SD of time per character	−0.68 (1.76)	−0.22 (−0.40, −0.03) ∗	.02
DEM-V score	0.54 (7.75)	Time per character	−3.07 (2.76)	0.17 (0.05, 0.28) ∗∗	.007
		Missing stroke	−1.84 (13.63)	−0.73 (−1.28, −0.19) ∗	.01
		HAC	1.48 (6.09)	0.34 (0.05, 0.64) ∗	.03
DEM-R	−0.11 (0.27)	Average pressure	29.46 (139.85)	−182.86 (−356.49, −9.23) ∗	.04
COMPS	0.82 (1.03)	Ground time	−32.10 (77.93)	−31.11 (−57.32, −4.90) ∗	.02
		Time per character	−3.07 (2.76)	−1.31 (−2.20, −0.42) ∗∗	.005
		SD of time per character	−0.68 (1.76)	−0.81 (−1.36, −0.26) ∗∗	.005
		Writing speed	0.96 (1.66)	0.59 (0.02, 1.16) ∗	.04
		Recognized character	−0.59 (8.51)	4.73 (2.16, 7.30) ∗∗∗	<.001
		Reversed stroke	−0.85 (6.60)	2.33 (0.08, 4.58) ∗	.04
		Wrong stroke sequence	−1.74 (12.05)	−5.74 (−9.55, −1.93) ∗∗	.004

Adjusted for gender; ∗<0.05, ∗∗<0.01, ∗∗∗<0.001.

### 3.4 Parents’ feedback

Table 6 shows parents' feedback after the intervention programme. The score for item 3 and 6 was 2.8 out of 5. Other items had a score over 3 out of 5, showing that in general the parents were able to better understand the handwriting problems of their children rather than just focusing on the speed and accuracy using the SHARP system. Some commented that they knew better the underlying reasons of their children's handwriting difficulties. Most parents reported improvements in their children's handwriting after the intervention. Item 7 was scored the highest, suggesting that the parents would recommend other students to participate in the SHARP assessment and intervention programme. There was no significant difference between Group A and Group B in terms of the parents' feedback.

**Table 6:** Parents’ feedback after the intervention programme.

Items		Total	Group A	Group B	
		Mean (*SD*)			*p*
1	Is the SHARP report easy to understand?	3.00 (0.64)	2.94 (0.44)	3.07 (0.83)	.59
2	With the SHARP assessment and report, I am able to understand the handwriting problem of my children.	3.10 (0.66)	2.94 (0.68)	3.29 (0.61)	.15
3	With the SHARP assessment and report, I know how to train my children's handwriting ability at home.	2.80 (0.85)	2.69 (0.95)	2.93 (0.73)	.43
4	After the intervention programme, I am able to understand the handwriting problem of my children.	3.13 (0.68)	2.94 (0.68)	3.36 (0.63)	.09
5	After the intervention programme, I know how to train my children's handwriting ability at home.	3.00 (0.79)	3.06 (0.77)	2.93 (0.83)	.65
6	After the intervention programme, my anxiety and pressure of facilitating my children's learning are alleviated.	2.80 (0.66)	2.75 (0.58)	2.86 (0.77)	.67
7	I would recommend to the school to let other students participate in the SHARP assessment and intervention programme.	3.27 (1.23)	3.19 (1.60)	3.36 (0.63)	.70

## 4 Discussion

This study evaluated a group-based sensorimotor intervention to improve Chinese handwriting in children aged 7–9 years. Results showed that the 8 sessions of intervention appeared to improve the handwriting performance of those students presented with handwriting and sensorimotor difficulties. The study showed the results of the handwriting process, motor control, and visual perceptual skills in participants with handwriting problems. The study also compared the effects of the groups with two different intervention durations. It is found that there was no significant difference between the two groups in terms of the improvement of handwriting and sensorimotor performance, except handwriting time per character, however, a longer training programme appears to be superior in helping these students in handwriting compared to those with more frequent training sessions per week but with a short duration. From the evidence of neuroscience literature, specificity (associated handwriting practice) and intensity are critical elements in inducing neuroplastic change [16]. While handwriting is a complex ability that needs to have a good integration of cognition, sensory, and motor output. Thus, it would take time to make a change in students’ handwriting performance. Moreover, according to the theory of motor learning, a distributed practice involving alternating tasks with rests is considered more suitable for motor learning than a mass practice with little rest [16].

Participants showed significant improvement in the handwriting process and motor skills. During the eight sessions of the intervention programme, participants have the opportunities to manipulate objects by using radial three fingers in tearing paper, rolling coins, and spinning pens. Children with the improved fine motor function will develop a more mature pencil grip (tripod grip), enabling a more stable gripping force and patterns with efficient adjustment. Through strengthening children's motor control and muscle coordination, the ground time for movement execution could be reduced, as shown by the results of this study [9,28,29]. This aligns with previous studies reassuring significant associations between in-hand manipulation skills and handwriting speed [9,11]. For the between-group comparison, Group A outperformed Group B in terms of improving writing time per character. The training sessions lasted for 4 weeks in group A while the training sessions only lasted for 2 weeks in group B, although the total number of training sessions remained the same. Longer training duration might allow more time for the participants to build up the fine motor and handwriting skills in Group A, and parents might be engaged more in the home training programme to encourage their children to write better.

Apart from motor skills, the improvement of visual perceptual skills also facilitates children's handwriting process. Better visual perceptual skills are acquired for quicker and more precise processing of visual information through the intervention. For instance, matching games can improve visual memory. The improved visual memory facilitates children to recall the orthographic structures of Chinese characters more efficiently, reducing air time and ground time for recalling character structures during Chinese handwriting. Our results are consistent with a previous study conducted by Poon and colleagues [5] who implemented a computerized visual perception program for children with handwriting difficulties. Remarkable improvements in visual perceptual skills and handwriting speed with shorter ground and air time were shown. Oculomotor control is another crucial visual component in handwriting as most individuals visually track their ongoing work. In the intervention, completing oculomotor worksheets with static posture (standing or sitting) could train students' visual pursuit to trace lines of digits and fill colors. The warm-up activities can optimal student's arousal levels via different sensations input and train children's core strength and endurance, facilitating them to maintain proper sitting posture. The improved core control enables maintenance of their heads at a centered position, contributing to more efficient visual pursuit. This could reduce the time needed for copying and increase handwriting speed.

It was also noted that there were variations among parents from the two groups. Some parents were more motivated to engage their children in home training after each session, but some were more passive. It might be possible that some parents attribute children's handwriting difficulties to their misbehaviors, including inattentiveness or laziness. Therefore, parental education will enhance their awareness of children's handwriting difficulties and underlying deficits, facilitating early identification of specific learning needs, attention deficit hyperactivity disorder (ADHD), and dyslexia in children. In particular, children with ADHD and dyslexia are found to manifest copying difficulties more commonly than typically developing peers, including slower handwriting speed and more copying errors, which underpin their immaturity of perception-motor skills [12,28,29].

Through applying SHARP for screening and assessment, children's handwriting problems could be reflected more accurately. Thus, parents could better understand their children's problems, which could provide better remediation and support to them. Home programs of sensorimotor training or incorporating computerized hardwiring training software using tablet can further be included by strengthening parents' involvement and participation in the training [30]. Parents play an essential role in monitoring their children's training progress at home and providing positive reinforcements with verbal praise, emotional support, and encouragement to enhance their children's motivation and cooperativeness. From this study, it was found that those parents engaged in the home training programme for a longer time, i.e., in Group A, the outcomes appeared more positive. Therefore, if parents are trained on sensorimotor activities as alternatives to conventional drilling for home programs, it could further enhance the outcomes. Furthermore, with proper parental coaching and support, children will have more home practices for developing sensorimotor skills and hence better handwriting performance.

There were several limitations to the study. First, a small sample size and a small age range of the children included in this study reduced the statistical power to detect a small effect, so the effect of intervention may be underestimated. A larger sample size is needed to investigate the effectiveness of the intervention in the future. Although convenience sampling has a higher possibility to cause biased samples, it was adopted due to social restrictions during the COVID-19 pandemic. There were restrictions on entries to schools and social gatherings, limiting our subject recruitment and face-to-face group sessions. As the handwriting and sensorimotor performance may continue to improve during and after the intervention, interim assessments and follow-ups are expected to monitor the progress and capture the long-term effect of the intervention. Due to covid, it remained difficult to have a control group for comparison. Thus, the time effect of the interventions could be explained by the maturity or practice effect. Future research will incorporate a control group to test the intervention effect. Nevertheless, this study provides an important groundwork to conduct a randomized controlled trial in future research. Parents' involvement in training their children is vital to the success of training. However, in this study, the parents were only encouraged to conduct the home programme. In future studies, parents’ involvement and participation could be documented through daily log or feedback so that their efforts could be measured as a mediator of the results.

## 5 Conclusions

The sensorimotor intervention programme appeared to improve the Chinese handwriting performance of children with borderline handwriting difficulties. Significant improvement was shown in increased handwriting speed after the intervention with improved fine motor control and visual perceptual skills. A lower treatment frequency with lengthened intervention period might be more helpful in improving Chinese handwriting, whereas an intensive intervention also appears to be efficient in enhancing students’ Chinese handwriting performance. The involvement of parents in conducting a longer home training programme would further strengthen the outcomes of handwriting. Further research studies could be conducted to have a larger sample size, a longer follow-up, and a control group in order to prove its efficacy.

## Funding statement

Leung Howard W.H. was supported by Innovation and technology fund [ITT/003/20GX].

## Declaration of competing interest

The authors declare that they have no known competing financial interests or personal relationships that could have appeared to influence the work reported in this paper.
