# Supplementary material for: Evaluation of a group-based sensorimotor intervention programme to improve Chinese handwriting of primary school students
Source: Heliyon. 2022 Dec 23;9(2):e12554. doi: 10.1016/j.heliyon.2022.e12554 (PMC9932709; doi:10.1016/j.heliyon.2022.e12554)
Supplement: Multimedia component 1 [file mmc1.pdf]

書寫訓練問卷

1. 請問您覺得智能書寫板的報告簡單易懂？

   (4: 非常同意/ 3:同意/ 2: 間中/ 1: 不同意/ 0: 非常不同意)

2. 通過智能書寫板評估及報告，我能清楚了解小朋友的書寫問題？

   (4: 非常同意/ 3:同意/ 2: 間中/ 1: 不同意/ 0: 非常不同意)

3. 通過智能書寫板評估及報告，我知道如何在家訓練小朋友書寫能力？

   (4: 非常同意/ 3:同意/ 2: 間中/ 1: 不同意/ 0: 非常不同意)

4. 透過參與書寫訓練小組，我能清楚了解小朋友的書寫問題？

   (4: 非常同意/ 3:同意/ 2: 間中/ 1: 不同意/ 0: 非常不同意)

5. 透過參與書寫訓練小組，我知道如何在家訓練小朋友書寫能力？

   (4: 非常同意/ 3:同意/ 2: 間中/ 1: 不同意/ 0: 非常不同意)

6. 透過參與書寫訓練小組，緩解了我在協助小朋友學習時的焦慮與壓力?

   (4: 非常同意/ 3:同意/ 2: 間中/ 1: 不同意/ 0: 非常不同意)

7. 我會建議學校讓所有學生進行智能書寫板測試？

   (4: 非常同意/ 3:同意/ 2: 間中/ 1: 不同意/ 0: 非常不同意)

Handwriting training questionnaire

1. Is the smart handwriting recognition platform (SHARP) report simple and easy to understand?

(4: Strongly agree / 3: Agree / 2: Sometimes / 1: Disagree / 0: Strongly disagree)

2. Through the smart handwriting recognition platform (SHARP) report, I can clearly understand my child's handwriting problem.

(4: Strongly agree / 3: Agree / 2: Sometimes / 1: Disagree / 0: Strongly disagree)

3. Through the smart handwriting recognition platform (SHARP) report, I know how to train my child's writing ability at home.

(4: Strongly agree / 3: Agree / 2: Sometimes / 1: Disagree / 0: Strongly disagree)

4. After the Handwriting Training Group, I can clearly understand my child's handwriting problem.

(4: Strongly agree / 3: Agree / 2: Sometimes / 1: Disagree / 0: Strongly disagree)

5. After the Handwriting Training Group, I know how to train my child's writing skills at home.

(4: Strongly agree / 3: Agree / 2: Sometimes / 1: Disagree / 0: Strongly disagree)

6. The Handwriting Training Group has relieved my anxiety and pressure of helping my child in handwriting.

(4: Strongly agree / 3: Agree / 2: Sometimes / 1: Disagree / 0: Strongly disagree)

7. I would recommend the school to let all students take the smart handwriting recognition platform (SHARP).

(4: Strongly agree / 3: Agree / 2: Sometimes / 1: Disagree / 0: Strongly disagree)
