# Supplementary material for: Evaluation of a group-based sensorimotor intervention programme to improve Chinese handwriting of primary school students
Source: Heliyon. 2022 Dec 23;9(2):e12554. doi: 10.1016/j.heliyon.2022.e12554 (PMC9932709; doi:10.1016/j.heliyon.2022.e12554)
Supplement: Multimedia component 3 [file mmc3.pdf]

小學生書寫能力評估

學生需知

1. 請在本頁及方格紙上填上所需資料。
2. 當測試員指示測試開始時，方可翻開背頁開始進行測試。
3. 背頁將印有 90 個中文字，測試者須由***左至右***，***上至下***抄寫。
4. 測試者須將所有中文字***盡快而清晰地*** 抄寫在所提供的方格紙上，不得在方格外書寫。
5. 測試過程中測試者如果寫錯字，***不用刪去該字或重抄***，直接抄寫下一個字。
6. 測試以***繁體字***進行，不能寫簡體字。
7. 如有任何問題，須於測試開始前向測試員詢問。測試開始後，將不能暫停或突然終止測試。

工	出	那	到	要	高	球	痛	境
不	奶	作	定	重	院	圈	道	德
文	永	但	些	相	留	集	想	影
支	在	你	明	度	部	最	意	增
民	有	似	和	穿	得	等	新	熟
主	如	弟	其	是	情	就	算	趣
包	考	汽	居	香	都	然	精	辦
台	吃	育	查	家	教	象	演	器
母	希	易	客	能	做	喝	察	整
警	我	的	美	特	常	越	歌	餐
