# Supplementary material for: Evaluation of a group-based sensorimotor intervention programme to improve Chinese handwriting of primary school students
Source: Heliyon. 2022 Dec 23;9(2):e12554. doi: 10.1016/j.heliyon.2022.e12554 (PMC9932709; doi:10.1016/j.heliyon.2022.e12554)
Supplement: Multimedia component 2 [file mmc2.pdf]

編號:________________________

小學生書寫能力測試

編號:		性別：	男 / 女
學校:		慣用手:	左 / 右
年級:		評估日期:	
出生日期:	年 月		

這項測試主要評估小學生中文書寫的準確度與速度。

